# Sirt6 deficiency results in progression of glomerular injury in the kidney

**DOI:** 10.18632/aging.101214

**Published:** 2017-03-28

**Authors:** Wen Huang, Hua Liu, Shuang Zhu, Michael Woodson, Rong Liu, Ronald G. Tilton, Jordan D. Miller, Wenbo Zhang

**Affiliations:** ^1^ Department of Healthcare, Qianfoshan Hospital Affiliated to Shandong University, Jinan, China; ^2^ Department of Ophthalmology, University of Texas Medical Branch, Galveston, TX 77555, USA; ^3^ Center for Biomedical Engineering, University of Texas Medical Branch, Galveston, TX 77555, USA; ^4^ Sealy Center for Structural Biology and Molecular Biophysics, University of Texas Medical Branch, Galveston, TX 77555, USA; ^5^ Department of Internal Medicine, University of Texas Medical Branch, Galveston, TX 77555, USA; ^6^ Neuroscience and Cell Biology, University of Texas Medical Branch, Galveston, TX 77555, USA; ^7^ Department of Surgery, Mayo Clinic, Rochester, MN 55905, USA

**Keywords:** Sirt6, aging, glomerular injury, podocyte

## Abstract

Aging is associated with an increased incidence and prevalence of renal glomerular diseases. Sirtuin (Sirt) 6, a nicotinamide adenine dinucleotide (NAD)-dependent histone deacetylase, has been shown to protect against multiple age-associated phenotypes; however it is unknown whether Sirt6 has a direct pathophysiologic role in the kidney. In the present study, we demonstrate that Sirt6 is expressed in the kidney and aging Sirt6-deficient mice exhibit renal hypertrophy with glomerular enlargement. Sirt6 deletion induces podocyte injury, including decreases in slit diaphragm proteins, foot process effacement, and cellular loss, resulting in proteinuria. Knockdown of Sirt6 in cultured primary murine podocytes induces shape changes with loss of process formation and cell apoptosis. Moreover, Sirt6 deficiency results in progressive renal inflammation and fibrosis. Collectively, these data provide compelling evidence that Sirt6 is important for podocyte homeostasis and maintenance of glomerular function, and warrant further investigation into the role of Sirt6 in age-associated kidney dysfunction.

## INTRODUCTION

Renal glomerular diseases, including glomerular hypertension, diabetic nephropathy, and focal segmental glomerulosclerosis (FSGS), affect public health [1]. Aging significantly increases the incidence and prevalence of these diseases. Podocyte structural changes and cellular loss, glomerular basement membrane thickening, glomerular enlargement, and glomerulosclerosis are all associated with aging [2]. Investigators have explored aging-related pathways in the development of glomerular diseases, and results of these studies have led to the identification of a critical role for Klotho, a well-known anti-aging molecule, in sup-pressing renal NF-κB activation and inflammation [3], inhibiting proteinuria and decreasing renal fibrosis [4], and attenuating renal hypertrophy and glomerular injury [5]. These results suggest that reduction of Klotho level during aging may contribute to renal diseases such as diabetic nephropathy. Additionally, overactive mTOR is linked to aging and diabetic nephropathy, and mTOR inhibitors are beneficial for a number of renal diseases [6-8]. Progress in the field of molecular mechanisms contributing to aging has increased exponentially in recent years, with the impact of many of these signaling pathways on renal function remaining poorly understood.

Sirtuins (Sirts) are members of a highly conserved family that share homology with yeast Sir2 protein, a nicotinamide adenine dinucleotide (NAD)-dependent histone deacetylase regulating life span of yeast [9]. Among seven mammalian Sirts, Sirt6 most closely resembles yeast Sir2 regarding its intracellular location, function, and animal phenotype caused by Sirt6 deletion [9, 10]. Sirt6 is a histone H3 lysine 9 (H3K9) and H3K56 deacetylase that represses the transcription activities of several transcription factors involved in aging and inflammation [11-17]. It also promotes DNA repair, prevents genomic instability and maintains glucose homeostasis [18]. These multiple functions of Sirt6 uniquely position it as a key anti-aging molecule. Correspondingly, loss of sirt6 results in a gross phenotype that resembles premature aging while overexpression of sirt6 enhances life span [9, 18]. While Sirt6 has been found to play critical roles in aging-related diseases such as cancer, bone loss, cardiovascular disease and neurodegenerative diseases [14, 17-21], the specific role of this enzyme in renal homeostasis and function is unknown.

In this report, we demonstrate that aging Sirt6-deficient mice exhibit renal hypertrophy, with glomerular enlargement. Sirt6 deletion leads to chronic inflammation and fibrosis in the kidney of these mice. We further show that the absence of Sirt6 results in proteinuria and podocyte depletion in mutant mice, which may be caused by podocyte foot process effacement and apoptosis. We conclude that Sirt6 is important for podocyte homeostasis and is a critical enzyme involved in the maintenance of renal function.

## RESULTS

### Sirt6 is functionally expressed in the mouse kidney

**Figure 1:**
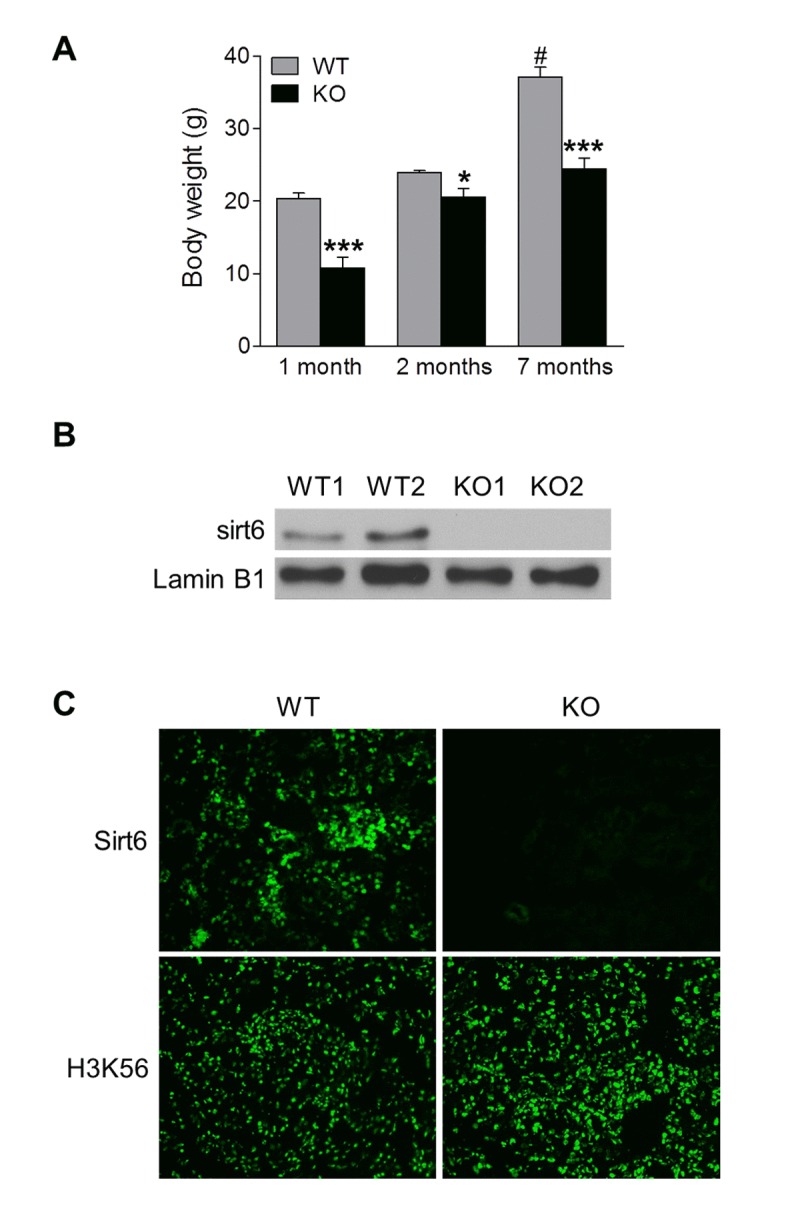
Functional Sirt6 is expressed in mouse kidney (**A**) Body weight of Sirt6 KO mice and their WT littermates was quantified at various ages. Data are presented as mean ± SEM; n=8-9; *P<0.05, ***P < 0.001 compared with relevant WT mice; ^#^P<0.05 compared with 1 month old WT mice. (**B**) Nuclear protein was extracted from the kidneys of WT and Sirt6 KO mice and Sirt6 protein expression was assessed by Western blot analysis. Lamin B1 served as loading control. (**C**) Immuno-fluorescence of Sirt6 and H3K56 in kidney cryosections from WT and Sirt6 KO mice.

To study the potential role of Sirt6 in the kidney, we generated Sirt6 global knockout (Sirt6^−/−^) mice on a C57BL6/129svJ mixed background. Consistent with previous reports [14], we found that some of the Sirt6^−/−^ mice died around 4 weeks of age, while the remainder of the Sirt6^−/−^ mice survived after weaning but died within 1 year. Analysis of the surviving mutant mice indicated that they had a lower body weight and size compared to WT littermates (Fig. 1A and Supplementary Fig. 1C). To confirm the absence of Sirt6 in the kidney of Sirt6^−/−^ mice, we examined Sirt6 expression by Western blot, and observed that abundant Sirt6 protein was present in the kidney extract from WT mice, whereas its expression was absent in that from Sirt6^−/−^ mice (Fig. 1B). In addition, immunohistochemistry demonstrated that Sirt6 staining was present in renal cryosections of WT mice but was absent in those from Sirt6^−/−^ mice (Fig. 1C, upper panel). Furthermore, the level of acetylation of H3K56, which is a substrate of Sirt6 [18], was significantly increased in Sirt6 deficient kidney compared to WT kidney (Fig. 1C, lower panel).

### Loss of Sir6 accelerates renal hypertrophy and progressive proteinuria

**Figure 2:**
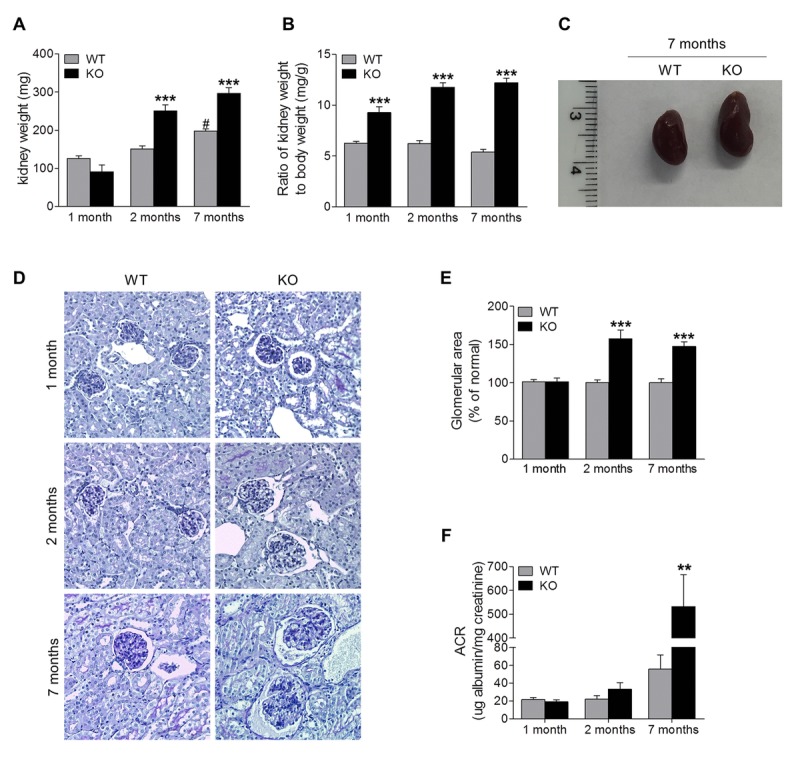
Sirt6 deficiency causes kidney hypertrophy and proteinuria (**A**, **B**) Kidneys from WT and Sirt6 KO mice were collected at 1, 2, 7 months of age. The weight of kidney was quantified (**A**) and the ratio of kidney weight to body weight was calculated (**B**). (**C**) Representative image of the kidneys from WT and Sirt6 KO mice at 7 months of age. (**D**) Periodic acid–Schiff staining of kidney paraffin section for histologic analysis. (**E**) Quantification of glomerular area (~15 glomeruli/section). (**F**) Urine was collected from WT and Sirt6 KO mice at indicated time points. Albumin and creatinine were measured by Albumin ELISA kit and Creatinine Colorimetric Assay Kit respectively, and ACR was calculated. Data are presented as mean ± SEM; n=6-9; **P<0.01, ***P <0.001 compared with relevant WT mice; ^#^P<0.05 compared with 1 month old WT mice.

To characterize the role of Sirt6 in the kidney, we analyzed changes in the renal phenotype of Sirt6^−/−^ mice compared with WT littermate controls at different ages. Kidney weight of Sirt6 deficient mice was slightly lower than that of WT mice at 1 month of age, but it was significantly higher than controls at 2 and 7 months of age (Fig. 2A). The ratio of kidney weight to body weight (KW/BW), a parameter for renal hypertrophy, was significantly higher in Sirt6^−/−^ mice than in their control littermates (Fig. 2B). Corresponding to the increase in kidney weight, kidney enlargement was visually observed in 2- and 7-month old Sirt6 null mice (data not shown and Fig. 2C). Histologically, PAS staining showed that glomerular area did not differ significantly between these two genotypes of mice at 1 month of age, but was increased in KO mice at 2 and 7 months of age (1.6-fold and 1.5-fold, respectively) (Fig. 2D and 2E). These results indicate Sirt6 is important for maintenance of renal phenotype.

To evaluate renal filtration function, levels of albumin and creatinine in urine samples were measured, and albumin/creatinine ratio (ACR), an index of excretion of serum albumin into the urine, was calculated (Fig. 2F). At 1 month of age, Sirt6 deficient mice were indiscernible from their WT littermates in the degree of proteinuria levels. However, at 2 months of age, Sirt6 KO mice developed slight proteinuria compared with its age-matched control, which was significantly increased by 7 months of age. The increasing proteinuria correlated with weight loss (Fig. 1A) and increased mortality after 7 months of age. These data indicated that Sirt6 deletion increased urinary albumin excretion, suggesting that Sirt6 is required for maintenance of normal renal function.

### Sirt6 deletion causes progressive podocyte dysfunction and depletion

**Figure 3:**
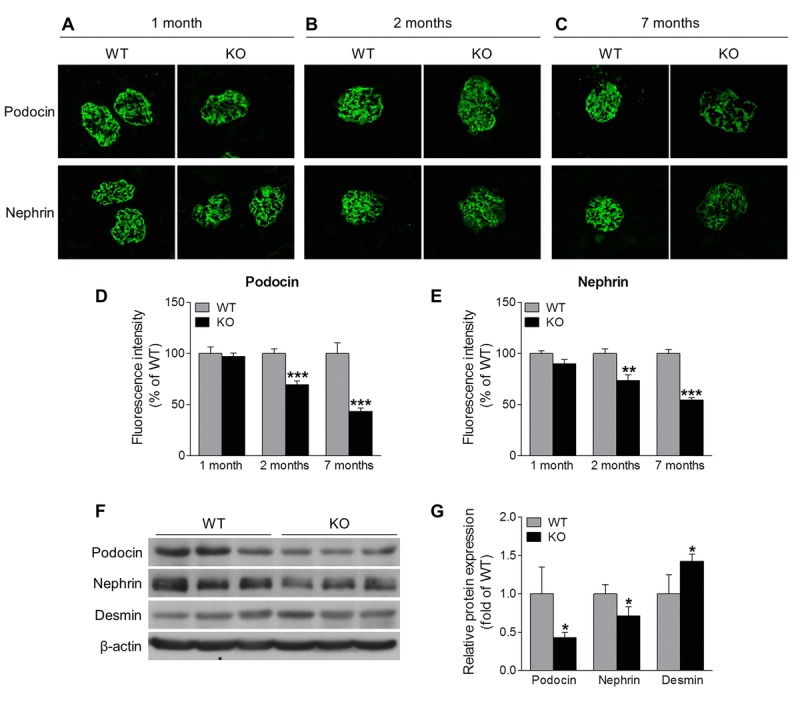
Sirt6 deletion induces podocyte injury (**A**-**C**) Immunostaining of podocin and nephrin (green) in the kidney cryosection from WT and Sirt6 KO mice at different ages. (**D**, **E**) Graphs represent the quantification of podocin and nephrin immunofluorescence. Data are presented as mean ± SEM. **P<0.01, ***P<0.001 compared with relevant WT mice. (**F**) The cortex of kidney from WT and Sirt6 KO mice was collected at 7 months of age and lysed, and podocyte slit diaphragm proteins (podocin and nephrin) and podocyte injury marker desmin were examined by Western blot analysis. (**G**) Graph represents the densitometry of podocin, nephrin and desmin expression normalized to β-actin. Data are presented as mean ± SEM; n=3; *p<0.05 compared WT.

**Figure 4:**
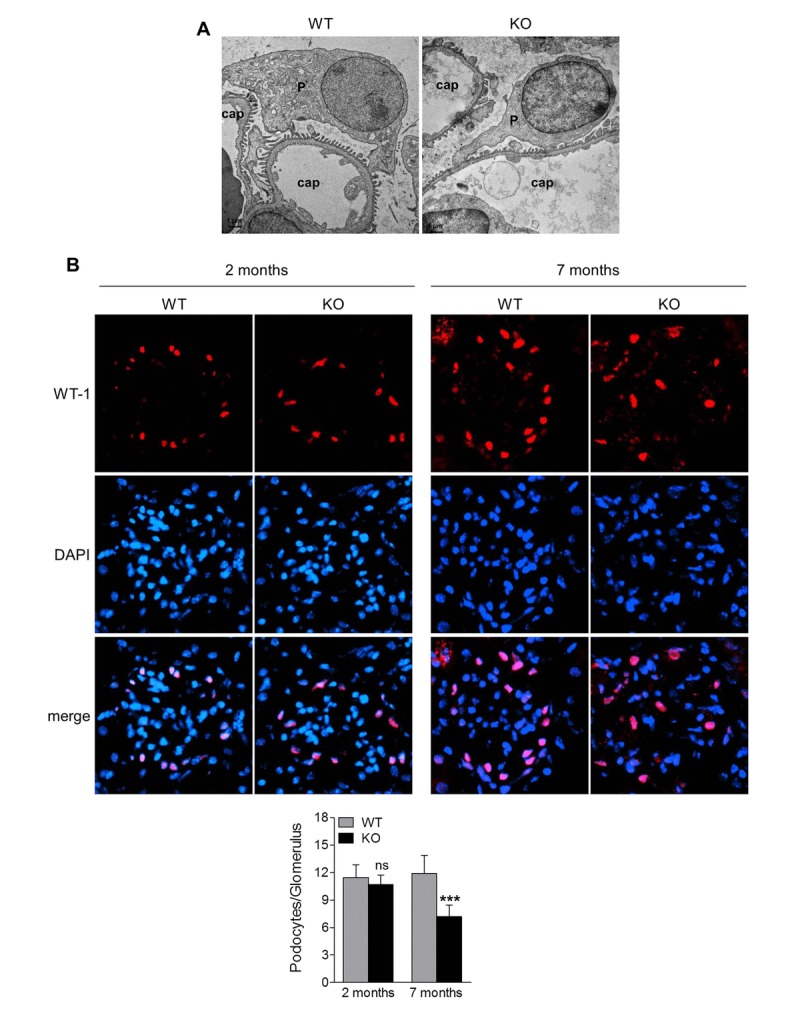
Sirt6 deletion results in podocyte foot process effacement and cellular loss (**A**) Ultrastructural analysis of the kidneys from WT and Sirt6 KO mice at 2 months of age by transmission electronic microscopy. P: podocyte; cap: capillary. (**B**) Kidney cyrosections from WT and Sirt6 KO mice at 2 and 7 months of age were stained with antibody against podocyte marker WT-1 (red) and images were taken by fluorescence microscopy. Blue: DAPI staining for nucleus. Podocyte numbers were counted randomly in 15 glomeruli/section and quantified. Data are presented as mean ± SEM. ***p<0.001 compared with 7-month old WT mice.

Podocytes are highly specialized and terminally differentiated glomerular cells with foot processes that interdigitate to form slit diaphragm, and that have an essential role in the maintenance of glomerular barrier function [22]. Podocyte injury is a clinical hallmark of proteinuria and glomerular disease. The increased proteinuria in Sirt6 KO mice prompted us to investigate the podocyte proteins, podocin and nephrin, that function to maintain the integrity of podocyte foot process slit diaphragm [22]. Immunofluorescence analyses revealed that the expression levels of podocin and nephrin were similar between 1-month old Sirt6^−/−^ and WT mice (Fig. 3A, 3D and 3E), while at 2 months of age, their expression levels were slightly lower in Sirt6^−/−^ vs WT mice (Fig. 3B, 3D and 3E). This observation correlates well with the onset of proteinuria in the KO mice. At 7 months of age, Sirt6-deficient mice demonstrated significantly less expression for these podocyte markers compared with WT mice (Fig. 3C-G), while the podocyte injury marker, desmin, was increased in kidneys of Sirt6 deficient mice vs WT controls (Fig. 3F and 3G). We evaluated podocyte structural alterations by transmission electron microscopy and observed that Sirt6-deficient podocytes displayed disorganization and effacement at 2 months of age (Fig. 4A). At this time point, the average number of podocytes per glomerular section, determined by Wilms' tumor 1 protein (WT-1) staining [23], was not significantly different between WT and Sirt6^−/−^ mice (Fig. 4B, left panel). But interestingly, the average number of podocytes was significantly decreased in Sirt6^−/−^ mice as they aged (Fig. 4B, right panel). Overall, Sirt6 knockout disrupts the slit diaphragm in podocytes, induces podocyte foot process effacement and final cellular loss. These findings are consistent with the marked increase in proteinuria in aged Sirt6 KO mice (Fig. 2F), suggesting the important role of Sirt6 in maintaining podocyte functional integrity.

### Sirt6 deletion induces podocyte apoptosis

**Figure 5:**
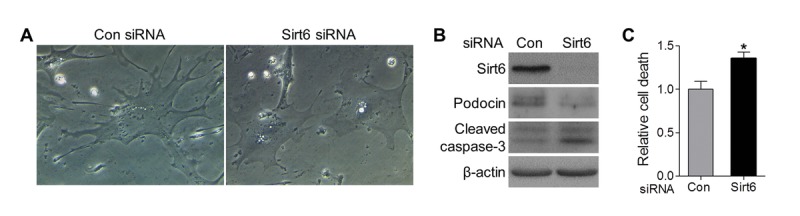
Sirt6 knockdown impairs foot process and induces podocyte apoptosis *in vitro* Primary murine podocytes were plated in a collagen coated 12-well plate at a density of 6x10^4^ cells/well and transfected with Sirt6 or control siRNA. (**A**) Two days after transfection, images of cell morphology change were taken by light microscope. (**B**, **C**) Three days after transfection, cells were harvested for western blot (**B**) and Cell death ELISA (**C**). Data are presented as mean ± SEM; n=3; *p<0.05 compared control siRNA.

Podocyte loss in aging Sirt6 KO mice could be caused by cell detachment with loss into the urinary space or by apoptosis. Prior evidence linking Sirt6 to cell survival led us to pursue apoptosis as a mechanism for podocyte loss in the kidney. To further evaluate the effect of Sirt6 on podocyte homeostasis, we specifically reduced Sirt6 protein levels using siRNA knockdown in cultured primary murine podocytes *in vitro*, and found that cells transfected with control siRNA exhibited normal structure with the characteristic arborized phenotype, whereas cells transfected with Sirt6 siRNA displayed morphological changes with loss of processes (Fig. 5A). Moreover, Sirt6 deletion increased podocyte apoptosis measured by the expression of activated caspase-3 (Fig. 5B) and cell death detection ELISA (Fig. 5C), suggesting Sirt6 function is associated with podocyte survival. Taken together, these data support the notion that Sirt6 deletion may induce podocyte apoptosis resulting in progressive podocyte depletion.

### Sirt6 deficiency causes chronic inflammation and fibrosis in the kidney

**Figure 6:**
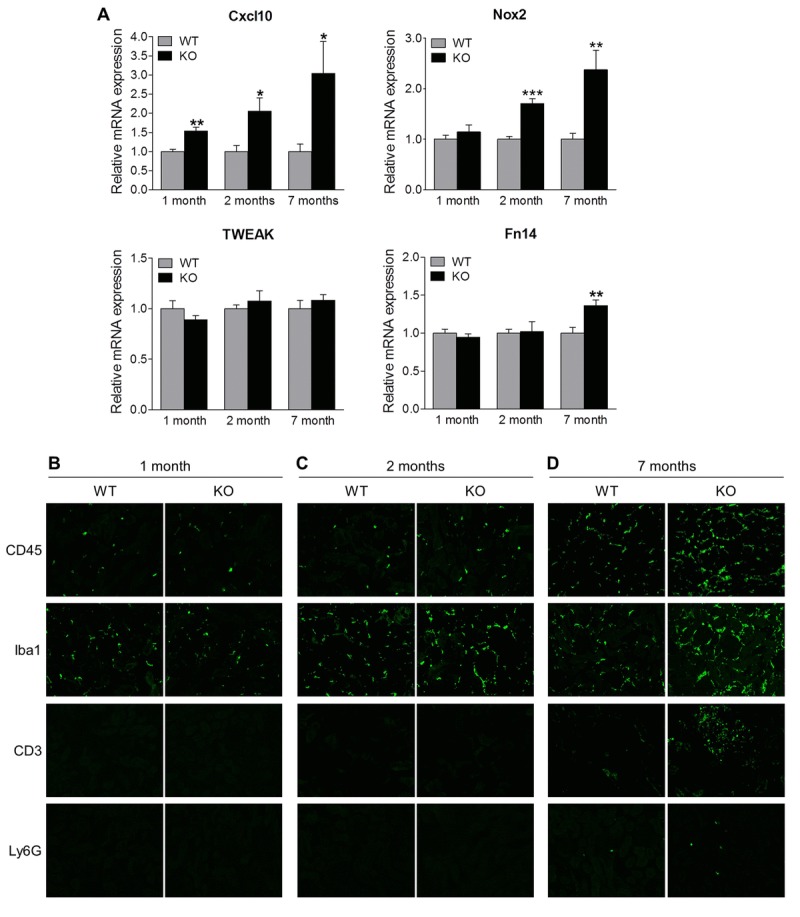
Sirt6 deficiency increases inflammation in the kidney (**A**) The cortex of kidney from WT and Sirt6 KO mice was collected at indicated time points and mRNA was extracted. The expression of Cxcl10, Nox2, Tweak and Fn14 mRNA was assessed by quantitative PCR. Data are presented as mean ± SEM; n=6; *p<0.05; **P<0.01; ***p<0.001 compared with relevant WT mice. (**B**-**D**) Kidney cyrosections from WT and Sirt6 KO mice at different ages were stained for leucocyte subtype markers CD45, Iba1, CD3 and Ly6G (green). Images were taken at 200X by fluorescence microscopy.

Recent studies have implicated inflammation in podocyte injury and fibrosis during chronic kidney disease [24-28]. Since Sirt6 plays a pivotal role in inflammation [12, 15, 20, 29, 30], we speculate that inflammation may also contribute to renal dysfunction in Sirt6 KO mice. Therefore we examined the role of Sirt6 in the renal inflammatory processes. To accomplish this, we assessed renal mRNA of inflammation-associated genes at our various time points and found that Sirt6 null mice exhibited increased transcription of Cxcl10 and Nox2 (Fig. 6A). These molecules have a critical role in recruitment of T cells and monocytes/macrophages, oxidative stress and renal injury [31-34]. The TWEAK/Fn14 pathway also contributes to renal inflammation and injury [35]. While TWEAK level was not changed, Fn14 expression was significantly increased in Sirt6 KO mice at 7 months of age. In parallel, immunostaining was performed to detect the infiltration of monocytes (CD45+), activated macrophages (Iba-1+), T cells (CD3+) and neutrophil (Ly6G+) (Fig. 6B-D). At 1 month of age, the immunoreactivity for CD45 or Iba1 was similar in both WT and Sirt6 KO kidneys (Fig. 6B), but by 2 months, KO mice showed higher immunoreactivity for CD45 or Iba1 than WT mice, suggesting Sirt6-deficient mice experienced increased infiltration of monocytes and activated macrophages in the kidney (Fig. 6C). At 7 months, Sirt6-deficient kidneys were characterized by intense Iba1 and CD45 immunoreactivity whereas WT kidneys exhibited only a moderate reactivity (Fig. 6D). T cells and neutrophils were present in small amounts in 7 month-old WT kidneys but dramatically increased in Sirt6 KO mice as shown in Figure 6B-D. These results indicate that Sirt6 deletion enhances aging-induced renal inflammation and suggest a critical anti-inflammatory role for Sirt6 in the kidney.

**Figure 7:**
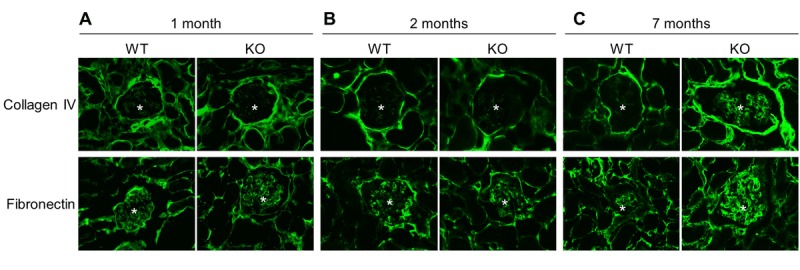
Sirt6 deficiency increases fibrosis Kidney cyrosections from WT and Sirt6 KO mice at different ages were stained with antibodies against collage type IV and fibronectin (green). Images were taken at 200X by fluorescence microscopy. Stars indicate glomeruli.

Since chronic inflammation often leads to fibrosis [24], we further examined renal fibrosis by immunofluorescence staining (Fig. 7). Analysis revealed that the deposition of collagen IV and fibronectin, well known makers of fibrosis, was similar in kidneys of Sirt6^−/−^ and WT mice at early ages (1 month and 2 months) (Fig. 7A and 7B) while the older, 7 month Sirt6 KO mice demonstrated enhanced glomerular presence of fibronectin and collagen IV (Fig. 7C). These results suggest an inhibitory function for Sirt6 in the development of renal fibrosis.

## DISCUSSION

Aging is an important risk factor for renal diseases, and while Sirt6 is increasingly recognized as a key anti-aging enzyme, its role in the kidney remains completely unknown. In this study, we have demonstrated that Sirt6 is expressed in the mouse kidney and its deletion causes progressive renal inflammation, glomerular hyper-trophy, loss of podocyte structure and function, and fibrosis. Assessment of renal function revealed severe proteinuria in aging Sirt6^−/−^ mice that was associated with podocyte dysfunction and depletion. Moreover, we demonstrated that Sirt6 deletion in podocytes *in vitro* led to reduction of foot process and cell apoptosis. These *in vivo* and *in vitro* findings demonstrate for the first time that Sirt6 has a crucial role in the maintenance of glomerular function, and consequently, may be an important, potentially new therapeutic target for renal diseases associated with aging.

In mammals, seven different sirtuins (Sirt1–7) have been identified with diverse cellular localizations [36]. Sirt1, Sirt6 and Sirt7 mainly reside in the nucleus, whereas Sirt3, Sirt4 and Sirt5 are predominantly localized in the mitochondria, and Sirt2 is primarily found in the cytoplasm. Sirtuins have been shown to be involved in age-related diseases, including diabetes, cardiovascular disease and neurodegeneration [36]. Recently, accumulating evidence indicates sirtuins are also involved in kidney disease. Specific deletion of Sirt1 in podocyte increases nephrotoxic serum-induced urinary albumin excretion and the severity of glomerular injury with marked podocyte injury, including actin cytoskeleton derangement [37]. Deleting Sirt1 in proximal tubules accelerates diabetes-induced proteinuria [38]. By contrast, Sirt1 activation by theobromine protects the diabetic kidney and may have therapeutic potential for diabetic nephropathy [39]. Sirt3 overexpression protects renal tubular epithelial cells against palmitate-induced lipotoxicity via anti-oxidative and anti-inflammatory mechanisms [40]. In contrast to the beneficial roles of Sirt1 and Sirt3, Sirt2 activity is linked to LPS-induced renal tubular inflammation and injury and decreased kidney function [41]. Of note, while Sirt1, Sirt2 and Sirt3 are involved in renal diseases, renal hypertrophy and fibrosis in mice deficient in these genes have not been reported. Our finding that loss of Sirt6 results in renal hypertrophy, proteinuria, inflammation, and fibrosis during aging and in the absence of additional exogenous stress provides the first demonstration of an important independent role for sirtuins in the maintenance of renal homeostasis and function. Overall, these studies highlight the critical functions of sirtuins in the kidney and warrant further exploration of pathophysiological roles of sirtuins and their downstream targets during renal aging and in the progression of renal diseases.

The precise mechanisms by which Sirt6 regulates renal homeostasis and function remain to be elucidated. Podocytes are highly specialized epithelial cells residing on the glomerular basement membrane (GBM) that play a central role in the maintenance of structure and function of the glomerular filtration barrier in the kidney [22]. Intact podocytes are characterized by foot processes that are connected by slit diaphragm proteins (such as nephrin and podocin). Injury to podocytes causing foot process effacement and final depletion is considered a key contributor in the development and progression of proteinuria and progressive glomerular injury [22]. From our observations, Sirt6 KO mice exhibit loss of slit diaphragm proteins and foot process effacement. While the total number of podocytes appeared similar in Sirt6 null mice and WT mice at an early age, over time, podocytes were progressively lost and by 7 months of age, podocyte numbers in KO mice were significantly reduced. Podocyte loss is likely the consequence of podocyte apoptosis as Sirt6 deletion in podocytes with siRNA significantly increased cell apoptosis. Thus, our *in vivo* and *in vitro* studies support the notion that Sirt6 is important in the regulation of podocyte homeostasis, contributing to normal renal physiology, although the underlying mechanism through which Sirt6 downregulates podocyte apoptosis remains unclear at this time.

In addition to its direct effects on podocytes, maintained expression of Sirt6 may protect against podocyte dysfunction and injury by regulating inflammation, a key player in the development and progression of chronic kidney diseases [24-28]. Sirt6 is known to inhibit inflammatory reactions by repressing the production of inflammatory molecules via interfering with key transcription factors in inflammation or by preventing endothelial cell dysfunction [42]. Loss of Sirt6 is associated with chronic inflammatory diseases while increasing Sirt6 has beneficial effects on attenuation of inflammation and tissue injury [20, 30, 43, 44]. Kidneys of Sirt6 KO mice exhibited hallmarks of renal inflammation, including upregulation of genes for pro-inflammatory molecules and oxidative stress, and progressive recruitment of monocytes/macrophages,

T lymphocytes and neutrophils. These leukocytes may secret inflammatory cytokines, chemokines and reactive oxygen species to further amplify renal inflammation as well as inducing podocyte damage [24]. Moreover, leukocytes secret fibrogenic cytokines that elicit renal fibrosis – a final common pathway leading to end-stage renal disease [24]. While our data provide the first evidence that Sirt6 negatively regulates renal inflammation, it is unclear how Sirt6 is involved in this process. Local renal cells, including podocytes, tubular epithelial cells and endothelial cells can express inflammatory molecules when losing Sirt6 expression. Alternatively, Sirt6 in leukocytes could participate in inflammation by regulating leukocyte recruitment and activation [15]. Future studies to specifically delete Sirt6 in podocytes, tubular cells, endothelial cells and leukocyte subtypes are necessary to determine which cell subtypes are primary contributors to the renal inflammation as observed in Sirt6 KO mice. Never-theless, regardless of the cellular source of Sirt6 in this process, our study suggests that modulation of Sirt6 activity could be beneficial for alleviation of renal inflammation.

In conclusion, the present study demonstrated that Sirt6 deletion induces glomerular changes, including inflammation and podocyte injury, that result in progressive podocyte depletion and proteinuria. These results warrant further exploration of Sirt6 in models of renal disease that could lead to the development of new therapeutic strategies to treat chronic renal disease by targeting Sirt6.

## MATERIALS AND METHODS

### Animals

All animal procedures were approved by the University of Texas Medical Branch Institutional Animal Care and Use Committee and in accordance with the procedures and practices of the National Institutes of Health Guide for the Care and Use of Laboratory Animals. Mice were maintained on a 12:12 light/dark cycle with ad libitum food and water. Sirt6 global knockout (Sirt6^−/−^) mice were generated using the Sirt6-floxed (Sirt6^flox/flox^) mice on a C57BL6/129svJ mixed background generously provided by Dr. Chuxia Deng (Supplementary Fig. 1) [45]. At various time points (1, 2 and 7 months of age), body weights and spot urine samples were collected, mice were euthanized, and kidneys were removed and weighed. A renal hypertrophy index was calculated as kidney weight/body weight (KW/BW, mg/g). A portion of the renal cortex was snap-frozen in liquid nitrogen for RNA and protein isolation. For morphological analysis and immunofluorescence staining, kidneys were either directly embedded in optimal cutting temperature compound (OCT, Tissue Tek; Sakura Finetek, Torrance, CA) or fixed in 4% paraformal-dehyde (PFA) and then embedded in OCT or paraffin.

### Morphologic analysis by PAS staining

Paraffin-embedded kidneys were cut into 3 μm-thick sections and stained with Periodic Acid-Schiff (PAS) kit (Sigma-Aldrich, St. Louis, MO) according to the manufacturer's instructions. Fifteen randomly selected glomeruli in the outer cortex of each kidney section were evaluated under light microscopy at 200X magnification. Glomerular areas were analyzed with ImageJ software by a masked observer.

### Immunofluorescence analyses

OCT-embedded kidneys were cut into 7 μm-thick sections for immunofluorescence staining. Sections were fixed in 4% PFA for 10 min, permeabilized with PBS containing Triton X-100 for 15 minutes at room temperature, blocked with blocking buffer (BioGenex, San Ramon, CA) for 1 hour, and then incubated with the following primary antibodies overnight at 4° C: rabbit anti-Sirt6, rabbit anti-Acetyl-Histone H3 (Lys56) from Cell Signaling Technology (Beverly, MA); goat anti-podocin, rabbit anti-nephrin and rabbit anti-Wilms' tumor (WT)-1 from Santa Cruz Biotechnology (Santa Cruz, CA); rat anti-CD45 and rat anti-Ly6G from BD PharMingen (San Diego, CA); rabbit anti-Iba1 from Wako (Osaka, Japan); rabbit anti-collagen type IV from EMD Millipore (Billerica, MA); rabbit anti-fibronectin from Abcam (Cambridge, MA); and anti-mouse CD3 FITC from eBioscience (San Diego, CA). Sections were then washed with PBS, and incubated with appropriate Alexa Fluor 488 or 594-labeled secondary antibodies (1:1000; Thermo Fisher Scientific, Lafayette, CO) at room temperature for 1 hour. Slides were mounted with mounting medium containing 4',6-diamidino-2-phenylindole (DAPI; Sigma-Aldrich), and images were taken with a fluorescence microscope. For each antibody, there were at least three mice per group. The mean fluorescence intensity of podocin and nephrin staining in the glomeruli was measured using ImageJ software.

### Measurement of urine creatinine and albumin

Urine albumin levels were measured by Mouse Albumin ELISA Kit (Bethyl Laboratories Inc., Montgomery, TX) and urine creatinine levels were assessed by Creatinine (urinary) Colorimetric Assay Kit (Cayman chemical, Ann Arbor, MI) according to the manufacturer's instructions. Urinary albumin-to-creatinine ratio (ACR) was calculated as: ACR (μg/mg) = urine albumin (μg/dL) / urine creatinine (mg/dL).

### Cell culture and transfection

Primary murine podocytes were purchased from PrimCells (San Diego, CA) and maintained at 37° C in RPMI 1640 medium (Corning, Corning, NY) containing 10% FBS (Hyclone, Logan, Utah). Podocytes were plated in a collagen-coated 12 well plate at a density of 6 x 10^4^ cells/well. Mouse Sirt6 siRNA (SiGenome SMARTpool, M-061392-00-0005; GE Dharmacon, Lafayette, CO) or negative control siRNA (Thermo Fisher Scientific, Waltham, MA) were transfected into podocytes using Lipofectamine2000 (Thermo Fisher Scientific) following manufacturer's instructions. Two days after transfection, podocyte morphology was imaged at 400X magnification using light microscopy (Leica Microsystems, Buffalo Grove, IL). For Western blotting or cell death ELISA, cells were harvested three days after transfection.

### Quantitative real-time PCR

Total RNA was extracted from renal cortex using Trizol (Life Technologies, Rockville, MD) according to the manufacturer's instructions, and then quantified and reverse-transcribed using High Capacity cDNA Reverse Transcription Kit (Life Technologies). cDNAs were amplified for 40 cycles using Power SYBR Green (Life Technologies) and gene-specific primers in a StepOnePlus PCR system (Life Technologies). Primer sequences for mouse transcripts were as follows: Hprt For-5'-GAA AGA CTT GCT CGA GAT GTC ATG-3'; Hprt Rev-5'-CAC ACA GAG GGC CAC AAT GT-3'; Cxcl10 For-5'-CAT CCC TGC GAG CCT ATC C-3'; Cxcl10 Rev-5'-CAT CTC TGC TCA TCA TTC TTT TTC A-3'; Nox2 For-5'- TCA AGA CCA TTG CAA GTG AAC AC-3'; Nox2 Rev-5'- TCA GGG CCA CAC AGG AAA A-3'; TWEAK For-5'-TGG GAA GAG ACC AAA ATC AAC A-3'; TWEAK Rev-5'- CCC AAT CTG GCG GTC GTA-3'; Fn14 For-5'-CTG GTT TTG GCG CTG GTT-3'; Fn14 Rev-5'-TCT CTC CGG CGG CAT CT-3'. Gene expression levels were calculated by comparison of Ct values (delta-delta Ct) using Hprt as the internal control.

### Western blot analysis

Renal cortex from 7 month-old WT and Sirt6 KO mice was homogenized in RIPA buffer (Thermo Fisher Scientific) supplemented with protease and phosphatase inhibitors (Roche, Indianapolis, IN). To detect Sirt6 expression, nuclei were extracted from renal cortex samples using CelLytic™ NuCLEAR™ Extraction Kit (Sigma-Aldrich). Equal quantities of protein from each sample were separated by 7.5% or 10% SDS-PAGE and transferred to PDVF membranes. After blocking with 10% milk, membranes were incubated with the following primary antibodies: rabbit anti-Sirt6, rabbit anti-Lamin B1, rabbit anti-cleaved Caspase-3 from Cell Signaling Technology; goat anti-podocin and rabbit anti-nephrin from Santa Cruz Biotechnology; rabbit anti-desmin and mouse anti-β-actin from Sigma-Aldrich. After washing, membranes were further incubated with the appropriate HRP-conjugated secondary antibodies (GE Healthcare Life Science, Piscataway, NJ). Subsequently, the protein samples were visualized by ECL2 western blotting substrate (Pierce, Rockford, IL), exposed to an X-ray film and developed with an X-ray processor. β-actin was used as loading control. Band intensities were analyzed using the NIH ImageJ software and protein expression was normalized to that of β-actin.

### Cell death detection ELISA

For the detection of apoptosis, a cell death detection ELISA^PLUS^ kit (Roche) was used [46]. Three days after transfection, podocytes were harvested in 60 μl lysis buffer, and 20 μl of the supernatant was assayed according to the manufacturer's instructions. Data were normalized to cell lysate protein concentration.

### Transmission electron microscopy

Mice were anesthetized by i.p. injection of a mixture of ketamine hydrochloride (100 mg/kg) and xylazine hydrochloride (10 mg/kg), and the chest was opened under a surgical microscope. After 50 μl heparin was injected into the left ventricle and circulated for 30 seconds, a butterfly needle was inserted into the left ventricle for perfusion of phosphate buffer (PB; 100 mM, pH 7.4) for 4 minutes at a speed of 1.5 ml/min. Left carotid artery and right carotid artery were then perfused for 2 minutes, respectively. Next, the perfusion process was repeated with 4% PFA in PB. After perfusion, kidney was removed and a portion of the renal cortex was cut into small pieces and fixed in 0.05 M cacodylate buffer containing 2.5% para-formaldehyde, 0.3% glutaraldehyde and 0.03% picric acid. Samples were then secondarily fixed with osmium tetroxide, en-bloc stained with uranyl acetate, dehydrated by successive concentrations of ethanol, and embedded in Polybed 812 resin. In semi-thin (1 micron) sections stained with methylene blue, non-sclerotic glomeruli were localized. Ultrathin sections were made of one or two glomeruli per tissue specimen and stained with lead citrate for transmission electron microscopy. Four to ten photographs, covering one or two glomerular cross-sections, were made with a JEOL 2100 transmission electron microscope (JEOL Ltd.; 3-1-2 Musashino, Akishima, Tokyo 196-8558, Japan). Images with a final magnification of approximately 2500× were obtained.

### Statistical analysis

The results are expressed as mean values±SEM. Statistical analysis was performed using GraphPad Prism Software. Group differences between were evaluated by Student's-test and one-way ANOVA followed by post hoc Student's t-test using the Student-Newman-Keuls method. A value of *P* < 0.05 was considered statistical significant.

## SUPPLEMENTARY MATERIALS FIGURE


