# Supplementary material for: Sirt6 deficiency results in progression of glomerular injury in the kidney
Source: Aging (Albany NY). 2017 Mar 28;9(3):1069–81. doi: 10.18632/aging.101214 (PMC5391219; doi:10.18632/aging.101214)
Supplement: Supplementary file 1 [file aging-09-1069-s001.pdf]

SUPPLEMENTARY MATERIAL

Generating Sirt6 global knockout mice

Sirt6-floxed (Sirt6flox/flox) mice on a C57BL6/129svJ mixed background were generously provided by Dr. Chuxia Deng, in which exons 2 and 3 of the Sirt6 gene were flanked by LoxP recombination sites [1]. C57BL/6J mice expressing Cre recombinase under the control of Tie2 promoter/enhancer (Stock No: 004128) were purchased from the Jackson Laboratory (Bar Harbor, ME). To generate Sirt6 global knockout (Sirt6$^{-/-}$) mice, Sirt6flox/flox mice were cross-bred with Tie2-Cre mice to generate Sirt6flox/flox•Tie2-Cre mice. Female Sirt6flox/flox•Tie2-Cre mice were further bred with male Sirt6flox/flox mice. The offspring were genotyped by tail genomic DNA. Because Tie2 promoter/enhancer drives expression of Cre-recombinase in the female germ line [2], it results in the global deletion of the maternally inherited floxed allele and some offspring were Sirt6$^{+/-}$. Sirt6$^{+/-}$ males were then crossed with Sirt6$^{+/-}$ females to obtain Sirt6$^{-/-}$ mice. Sirt6 floxed and null alleles were confirmed by PCR genotyping (Supplementary Figure 1A and 1B) using the following primers: F1-5'- GCT AAT GGG AAC GAG ACC AA-3', R1-5'-ACC CAC CTC TCT CCC CTA AA -3'; R3-5'-GCG TCC ACT TCT CTT TCC TG-3'. PCR products were 444 bp (F1 and R1 for Sirt6 floxed allele) and 524 bp (F1 and R3 for Sirt6 null allele), respectively.

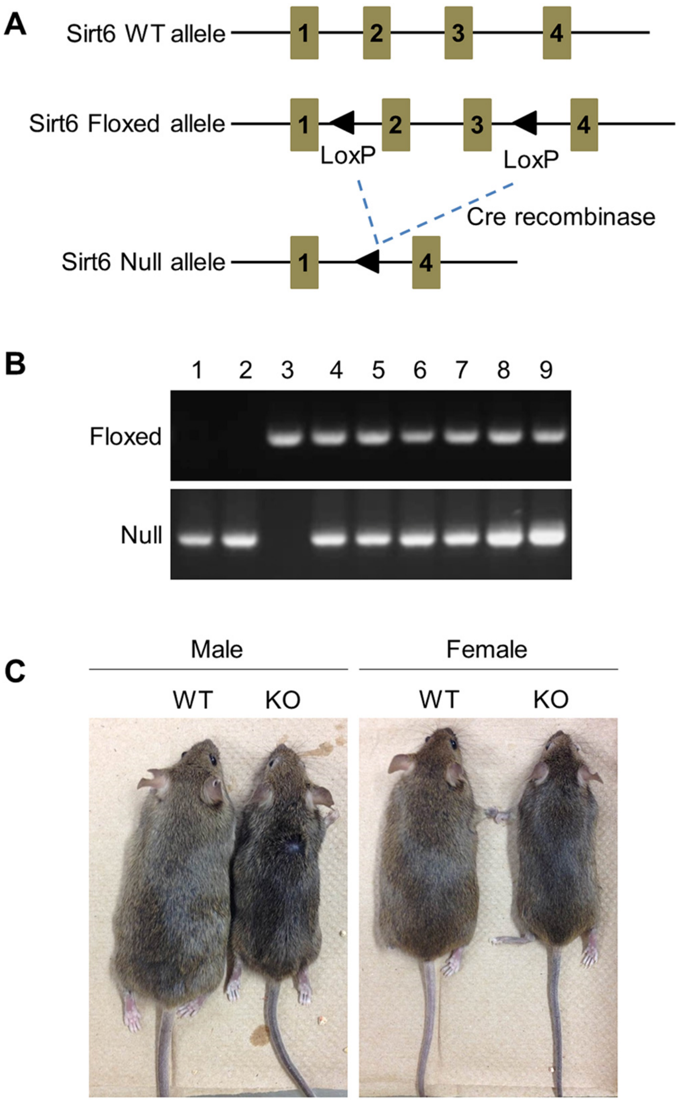

Supplementary Figure 1. Generation of Sirt6 KO mice. (**A**) Schematic representation of deletion of floxed Sirt6 exons (exons 2 and 3) by Cre expression. Black arrows: LoxP sites; gray bars: Sirt6 exons (**B**) Representative PCR genotying result of a litter or pups from breeding of a Sirt6$^{+/-}$ male with Sirt6$^{+/-}$ female. #1, 2: Sirt6$^{-/-}$; #3: Sirt6$^{+/+}$; #4:, 5, 6, 6, 8, 9: Sirt6$^{+/-}$. (**C**) Representative images of Sirt6 Control and KO mice at 7 months of age.

SUPPLEMENTARY REFERENCES

1. Xiao C, Kim HS, Lahusen T, Wang RH, Xu X, Gavrilova O, Jou W, Gius D, Deng CX: SIRT6 deficiency results in severe hypoglycemia by enhancing both basal and insulin-stimulated glucose uptake in mice. J Biol Chem. 2010; 285**:** 36776-36784.

2. de Lange WJ, Halabi CM, Beyer AM, Sigmund CD: Germ line activation of the Tie2 and SMMHC promoters causes noncell-specific deletion of floxed alleles. Physiol Genomics. 2008; 35**:** 1-4.
